# Potential of Lactoferrin Against the Radiation-Induced Brain Injury

**DOI:** 10.3390/cells14151198

**Published:** 2025-08-04

**Authors:** Marina Yu. Kopaeva, Anton B. Cherepov, Irina B. Alchinova, Daria A. Shaposhnikova, Anna V. Rybakova, Alexandr P. Trashkov

**Affiliations:** 1 National Research Center “Kurchatov Institute”, 1 Akademika Kurchatova sq., 123182 Moscow, Russia; 2 Institute of General Pathology and Pathophysiology, 8 Baltiyskaya St., 125315 Moscow, Russia

**Keywords:** lactoferrin, gamma-irradiation, survival rate, open field and elevated plus-maze tests, exploratory activity, brain and bone marrow, dentate gyrus of the hippocampus, proliferative activity

## Abstract

The purpose of this work was to study the effects of lactoferrin (Lf) on acute (days 3 and 15) and early-delayed (day 30) changes in the dentate gyrus of mouse hippocampus caused by whole-body gamma-irradiation. Male C57BL/6 mice received Lf (4 mg per mouse, i.p. injection) immediately after whole-body gamma-irradiation at a dose of 7.5 Gy from a ^60^Co source. The effect of Lf on mouse behavior was evaluated using “Open field” and “Elevated plus-maze” tests. The proportion of cells with DNA replication was determined by 5-ethynyl-2′-deoxyuridine incorporation (thymidine analog) and detected by a click reaction with azide Alexa Fluor 568. Lf treatment increased animal survival during the experiment (30 days), compensated for radiation-induced body weight loss, and prevented suppression of motor and exploratory activities. A pronounced anti-radiation effect of Lf on mouse brain cells has been demonstrated. A single injection of the protein allowed preserving 2-fold more proliferating cells and immature neurons in the dentate gyrus of the hippocampus of irradiated animals during the acute period of post-radiation injury development.

## 1. Introduction

Sources of ionizing radiation are now widely used in various fields. Humans can be exposed to radiation during professional activities or medical procedures, space exploration, or catastrophic accidents. In modern medicine, radiation therapy is one of the leading treatment methods for cancer patients that significantly increase their chances for recovery [1,2]. During radiation therapy and diagnostics, the patients are at risk of developing side effects such as changes in blood composition, immune suppression, neurological complications, and progressive cognitive dysfunction [3,4]. The range of reactions to radiation is determined by the radiation source, absorbed dose, duration of exposure, genetic features, and individual sensitivity of the organism [5]. Complications of radiation therapy can be divided by the time of their manifestation into acute (from several days to several weeks), subacute or early-delayed (from 1 to 6 months), and late (>6 months) [4,6].

Radiation-induced brain damage can lead to molecular, cellular, and functional disorders [7]. This is a continuous and dynamic process. Translational studies on laboratory animals have shown that radiation stimulates apoptosis and inhibits proliferation and neurogenesis in the dentate gyrus of the hippocampus in young adult rats and mice [8,9,10]. Late delayed reactions that occur several months or years after radiation therapy are progressive and irreversible [11]. Cognitive deficits are the second most important factor, after survival, affecting the quality of life of patients who have undergone radiation therapy [12]. The loss of nerve progenitor cells and suppression of neurogenesis in the subgranular zone of the dentate gyrus definitely contribute to the pathogenesis of cognitive impairment caused by radiation [10,11,13].

The search for effective and safe drugs for the prevention and pathogenetic therapy of radiation damage has been conducted for a long time and remains extremely relevant [14], because the current therapeutic options, although beneficial, produce side effects that can persist for a long time and affect the quality of life of patients.

Lactoferrin (Lf), a glycoprotein from the transferrin family, attracts our attention as a potential agent for early radioprotective therapy, because along with participation in diverse physiological processes, such as binding and transport of iron ions and immune and inflammatory responses [15,16], this protein exhibits radioprotective [17,18,19] and neuroprotective properties [9,20,21]. Lf is certified by U.S. Food and Drug Administration as a “generally recognized as safe” substance (GRAS) [22]. The safety and tolerability of Lf even at high doses were demonstrated in animal experiments [23,24] and clinical studies [25,26]. Lf exhibits high bioavailability upon oral, intranasal, intravenous, and intraperitoneal administration and has a wide range of molecular targets [27].

Here, we studied the effects of single Lf administration on acute and early delayed alterations in the dentate gyrus of mouse hippocampus caused by acute whole-body gamma-irradiation using a wide range of methods at the body, organ, tissue, and cellular levels.

## 2. Materials and Methods

The study was performed on 140 male C57BL/6 mice (age 2–2.5 months, body weight 20–28 g; SPF status) purchased from the Institute of Cytology and Genetics of the Russian Academy of Sciences (Novosibirsk, Russia). The animals were kept in standard laboratory cages (1285L; Techniplast, Buguggiate, Italy), 3–5 mice per cage, at controlled temperature of 22 ± 2 °C, air humidity 55 ± 10%, and 12/12 h dark–light regimen and had free access to food and water. All experimental procedures were performed between 9:00 a.m. and 6:00 p.m. All manipulations were carried out in accordance with Directive 2010/63/EU on the Protection of Animals Used for Scientific Purposes [28] and were approved by the Local Ethical Committee for Biomedical Research of the National Research Center “Kurchatov Institute” (protocol No. 02-4 of 20 June 2023).

We used Lf isolated from human colostrum (purity 97%; Lactobio LLC, Moscow, Russia).

### 2.1. Experimental Groups

**Figure 1:**
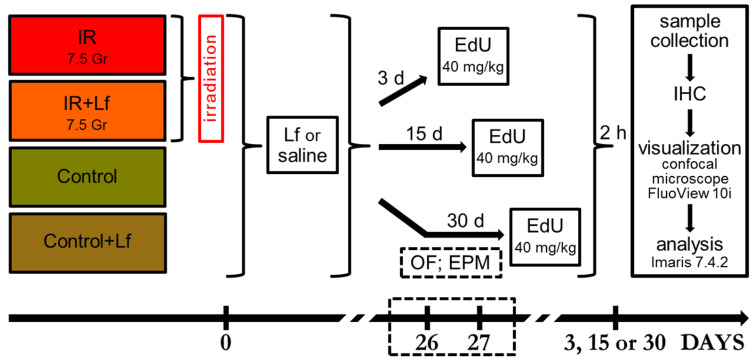
Timeline of the experiment. Animals from experimental groups were exposed to 7.5 Gy whole-body gamma-irradiation. Lf (i.p.; 4 mg/mouse) was administered immediately after irradiation/sham-irradiation (IR+Lf, Control+Lf). The open field (OF) and elevated plus-maze (EPM) tests were performed on days 26 and 27 after irradiation, respectively. Three, 15, or 30 days after irradiation, the mice were injected with a thymidine analog, 5-ethynyl-2′-deoxyuridine (EdU). Two hours after EdU administration, the mice were anesthetized, and the brain and bone marrow were sampled for immunohistochemical (IHC) analysis.

The mice were randomized into two experimental groups (Irradiation, IR, *n* = 44; Irradiation+Lf, IR+Lf, *n* = 44) and two control groups (Control, *n* = 26; Control+Lf, *n* = 26). Animals from the experimental groups were exposed to whole-body irradiation with gamma-rays from a ^60^Co source in a GUT-200M unit (National Research Center “Kurchatov Institute”, Moscow) at a dose of 7.5 Gy (dose rate 0.6 Gy/min). This dose causes serious changes in physiological and behavioral parameters in mice [19,29,30]. Immediately after exposure, the animals were injected with Lf (4 mg/mouse intraperitoneally in 0.3 mL of saline solution; IR+Lf group) or saline solution (IR group). The dose and the time of Lf administration were chosen based on our previous studies and reports of other authors [9,17,19]. Mice from the control groups (Control+Lf and Control) received injections of Lf and saline after sham irradiation. Figure 1 shows the design of the experiment.

### 2.2. Weighing

The animals were weighed before irradiation and then every 3 days throughout the experiment on an Adventurer Pro electronic scales (Ohaus, Pine Brook, NJ, USA), readability 0.01 g.

### 2.3. Open Field Testing

The open field test (OF) is a standard method for assessing spontaneous motor activity and behavior in rodents [31]. The model is based on the conflict of two motivations—instinctive drive to explore new environments and drive to avoid potential danger from open and brightly illuminated areas. OF was a round plastic arena with a diameter of 120 cm surrounded by 45 cm walls with an illumination level of 115 lux. Before placing each animal in the OF, the walls and the floor of the arena were cleaned with 70% ethanol. The arena was divided into the following zones: peripheral (a 10 cm wide ring adjacent to the arena wall), central (a circle with a diameter of 60 cm in the center of the arena), and intermediate (between the central and peripheral zones). Testing was performed on day 26 after irradiation (Figure 1), as described in detail in our previous report [19]. Each animal was placed in the center of the OF for 5 min to explore the arena, and then the behavior was recorded with an EthoVision XT 8.5 video recording system (Noldus Information Technology, Wageningen, The Netherlands).

The records were analyzed using EthoVision XT 8.5 software. The traveled path (total and in each zone), the duration of stay in the central, intermediate, and peripheral zones, the number of visits to the central zone, and the number of rearings were calculated. 

### 2.4. Analysis of Elevated Plus-Maze Behavior

Elevated plus-maze (EPM) test is one of the most sensitive tools for studying anxiety in rodents [32]. The maze was positioned at a height of 50 cm above the floor and consisted of two open (30 × 5 cm) and two closed (30 × 5 × 15 cm) arms extending from a central platform (5 × 5 cm) at an angle of 90°. The testing was performed on day 27 after irradiation (Figure 1). The mouse was placed on the central platform facing the open arm, and its behavior was recorded over 5 min with an EthoVision XT 8.5 video recording system (Noldus Information Technology, The Netherlands). Then, the mouse was returned to the home cage. The walls and the floor of the maze were cleaned with 70% ethanol after each test.

The records were analyzed using EthoVision XT 8.5 software. The path traveled (total and separately in the open and closed arms and in the center) was calculated. The time spent in open arms and the number of rearings and head dippings (head lowering over the edge of the open arm) were recorded manually. The entry into either arm was counted when the mouse had its body and four paws on the arm [33].

### 2.5. Sample Collection

On days 3, 15, or 30 after irradiation, the mice were intraperitoneally injected with a thymidine analog, 5-ethynyl-2′-deoxyuridine (EdU; 40 mg/kg; Lumiprobe, Moscow, Russia, 10540) [10,34] (Figure 1). Two hours after EdU administration, the animals were intramuscularly anesthetized with telazol (Zoetis, Girona, Spain) and rometar (Bioveta, Ivanovice na Hané, Czech Republic) in saline solution, and the blood was taken from the heart into 0.5 mL MiniCollect EDTA tubes (450530, Greiner Bio-One, Kremsmünster, Austria); then, transcardial perfusion with PBS (pH 7.4), and then with a 4% solution of paraformaldehyde (Sigma-Aldrich, St. Louis, MO, USA) in PBS, was performed. The brain was isolated and postfixed with a 4% solution of paraformaldehyde (Sigma-Aldrich, USA) in PBS at 4 °C for 24 h.

Bone marrow cells were routinely isolated from the femoral bones [35]. In brief, the femurs were excised and cleaned from the muscle tissue, and the epiphyses were removed. The bone was placed in a 0.5 mL microcentrifuge tube (1110-00; Scientific Specialties Inc., Lodi, CA, USA) with a hole in the bottom made with an 18 G needle, the cap was closed, and the tube was nested into a 1.5 mL microcentrifuge tube (1260-00; Scientific Specialties Inc., Lodi, CA, USA) and centrifuged in a Sigma 1–14 microcentrifuge (Sigma, Osterode am Harz, Germany) at 10,000× *g* for 10 s. The bone marrow pellet in the larger tube was suspended in 100 µL of PBS for further analysis.

### 2.6. Blood Tests

Complete blood count was performed on a DF50 automatic hematology analyzer (Shenzhen Dymind Biotechnology Co., Ltd., Shenzhen, China). The number of leukocytes, erythrocytes, platelets, and hemoglobin level were determined.

### 2.7. Immunocytochemical Analysis of Bone Marrow Cells

Bone marrow cells isolated from the mouse femoral bones (see Section 2.5) were suspended in 100 µL of PBS, fixed by adding 1200 µL of 4% paraformaldehyde (Sigma-Aldrich, USA) in PBS and left for 20 min at room temperature. The cells were precipitated by centrifugation at 500× *g* for 5 min, 500 µL of cooled PBS was added to the precipitate, and the samples were stored at 4 °C.

#### 2.7.1. EdU Staining

A 100 µL aliquot of the bone marrow cell suspension was twice washed with PBS. To reduce nonspecific staining and permeabilize the membranes, the cell suspension was incubated with 1% BSA (A7030, Sigma-Aldrich, USA) in 0.5% Triton X-100 (Sigma-Aldrich, USA) in PBS (0.5% TBS) for 30 min at room temperature. After double washing in 0.1% TBS, a click reaction with Alexa Fluor 568 azide (10 µM; Lumiprobe, Moscow, Russia, A5820) was carried out in the presence of 120 mM sodium ascorbate (Sigma-Aldrich, USA) and 6 mM CuSO_4_ (Sigma-Aldrich, USA) for 20 min at room temperature according to the Salic and Mitchison protocol [36]. Cell nuclei were poststained with Hoechst 33342 (1 µg/mL; Thermo Fisher Scientific, Rockford, Il, USA, 62249).

#### 2.7.2. Flow Cytometry

Bone marrow cells were analyzed on a BD FACSAria Fusion flow cytofluorometer (BD Life Sciences, San Jose, CA, USA) equipped with lasers with excitation wavelength spectrum from 355 to 640 nm. Emitted fluorescence was recorded in channels FL1 (598 nm, AF568) and FL2 (486 nm, Hoechst 33342). In each sample, 10,000 events were accumulated. FACSDiva 8.0.3 software (Becton Dickson, Franklin Lakes, NJ, USA) was used for data acquisition. The data were processed using FlowJo 10.5.3 software (Tree Star Inc., Ashland, OR, USA), and the percentage of EdU^+^ cells from all nucleated bone marrow cells was determined.

### 2.8. Immunohistochemical Analysis

#### 2.8.1. Brain Sections

For each animal (*n* = 5 term/group), one brain hemisphere was randomly selected. Serial sagittal sections (50 µm) were sliced on a Leica VT1200S vibratome (Leica, Nussloch, Germany) and transferred successively to six wells of a 24-well culture plate filled with PBS in such a way that each next slice in the same well was located at a distance of 300 µm from the previous one. Thus, six representative sets of 11–12 brain sections were prepared [37]. The sections were stored in PBS at 4 °C or in a cryoprotectant (glycerin, ethylene glycol, PBS, 1:1:2, *v*/*v*; all Sigma-Aldrich, USA) at −20 °C until staining.

For staining and counting, all sections containing the dentate gyrus of the hippocampus (*n* = 7–9) from one well were used [38].

#### 2.8.2. EdU Staining

The sections were washed 3 times with PBS to remove the cryoprotectant and then incubated with 5% normal goat serum (NGS; Abcam, Cambridge, UK, ab7481) in PBS with 2% Triton X-100 (Sigma-Aldrich, St. Louis, MO, USA) (2% TBS) for 1 h at room temperature to reduce nonspecific staining and permeabilize the membranes, washed with 0.2% TBS (3 × 10 min), and a click reaction was performed with Alexa Fluor 568 azide (10 µM; Lumiprobe, Moscow, Russia, A5820) in the presence of 120 mM sodium ascorbate (Sigma-Aldrich, USA) and 6 mM CuSO_4_ (Sigma-Aldrich, USA) for 20 min at room temperature according to the Salic and Mitchison protocol [36]. The sections were washed in 0.2% TBS (2 × 10 min) and then in PBS. Cell nuclei were poststained with Hoechst 33342 (1 µg/mL; Thermo Fisher Scientific, 62249).

#### 2.8.3. DCX and NeuN Staining

Prior to triple staining with EdU, DCX, and NeuN, the sections were first incubated in 10 mM citrate buffer (pH 6.0) at 95 °C for 10 min for antigen unmasking, washed with PBS (3 × 10 min), permeabilized in 2% TBS with 2.5% normal donkey serum (NDS; D9663, Sigma-Aldrich, USA) for 1 h at room temperature, and washed again in 0.2% TBS (3 × 10 min). Then, the sections were incubated with rabbit polyclonal antibodies to doublecortin protein (DCX) expressed by immature neurons (anti-DCX antibody; 1:1000, ab18723; Abcam) and mouse monoclonal antibodies to neuronal nuclear protein (NeuN) located in the nuclei and perinuclear cytoplasm of mature neurons (anti-NeuN antibodies, clone A60; 1:500, MAB377; Millipore, Temecula, CA, USA) in 0.2% TBS with 2.5% NDS (D9663, Sigma-Aldrich, USA) for 12–18 h at 4 °C and additionally 2 h at room temperature. After 3-fold washing with 0.2% TBS, the sections were incubated with donkey anti-rabbit antibodies labeled with Alexa Fluor 488 fluorophore (1:500, ab150073; Abcam), and donkey anti-mouse antibodies labeled with Alexa Fluor 647 fluorophore (1:500, A-31571; Invitrogen, Carlsbad, CA, USA) in 0.2% TBS for 2 h in the dark at room temperature. After 3-fold washing with 0.2% TBS, click reaction was performed with Alexa Fluor 568 azide (Lumiprobe, Moscow, Russia, A5820), as described above. The cell nuclei were poststained with Hoechst 33342 (1 µg/mL; Thermo Fisher Scientific, 62249).

For verifying specificity of immunostaining, the control sections were processed by the same protocol, but the primary antibodies were omitted.

#### 2.8.4. Immunofluorescence Analysis

Fluorescent brain sections were embedded in Fluoromount Aqueous Mounting Medium (F4680, Sigma-Aldrich, USA) under coverslips and digitized using a FluoView 10i confocal microscope (Olympus, Japan). The obtained images were analyzed using the Imaris 7.4.2 software package (Bitplane, St. Paul, MN, USA).

EdU- and DCX-immunoreactive cells in the dentate gyrus of the hippocampus were counted manually by an experimenter blind to the groups. One set of seven to nine sections on average, covering the extent of the dentate gyrus, was used [34,37]. The cell counts for each animal are expressed as mean number per representative section.

### 2.9. Statistical Analysis

The results were processed statistically using the GraphPad Prism 8.0.1 software package (La Jolla, CA, USA). Animal survival was evaluated using the Kaplan–Meier method (Gehan–Breslow–Wilcoxon test). For comparative analysis, parametric one-way ANOVA was applied followed by the Šidák *post hoc* test, or nonparametric Kruskal–Wallis ANOVA followed by a *post hoc* comparison using the Dunn test or the Mann–Whitney U test. The differences were significant at *p* < 0.05. The data are presented as the mean and standard error of the mean or as medians, quartiles, and minimum and maximum values.

## 3. Results

### 3.1. Treatment with Lf Increased the Survival Rate and the Mean Lifespan of Irradiated Mice During the Experiment (30 Days)

**Figure 2:**
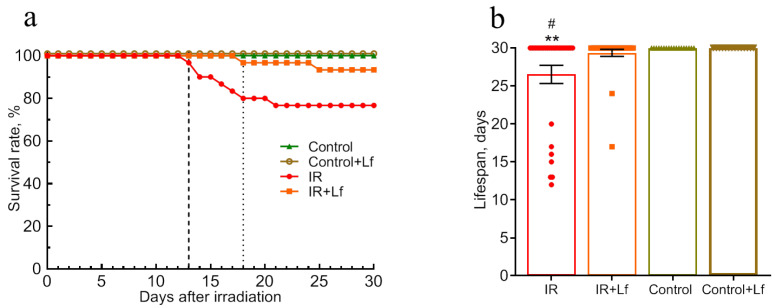
Effects of Lf on survival rate (**a**) and lifespan (**b**) of mice after gamma-irradiation. The survival rates (%) and lifespan (days) during the 30-day period after irradiation are presented. *n* = 30/group (for IR and IR+Lf), *n* = 14/group (for Control and Control+Lf). ** *p* < 0.01 in comparison with the Control group; ^#^ *p* < 0.05 in comparison with the IR+Lf group.

The effect of Lf on mouse survival and mean lifespan was studied by daily monitoring over 30 days after irradiation (IR, IR+Lf: *n* = 30/group). In the control groups (Control, Control+Lf: *n* = 14/group), no animal deaths were recorded during the experiment. In the IR and IR+Lf groups, the mice died starting from day 13 and day 18 after irradiation, respectively (Figure 2a, vertical dotted lines). It was found that administration of Lf increased the survival rate of irradiated animals from 76.7% (IR group) to 93.3% (IR+Lf group) (Figure 2a) and the mean lifespan during the experiment from 26.5 ± 1.2 (IR) to 29.4 ± 0.5 (IR+Lf) days (*p* = 0.024) (Figure 2b).

### 3.2. The Positive Effect of Lf on the Dynamics of Body Weight Recovery in Irradiated Mice

**Figure 3:**
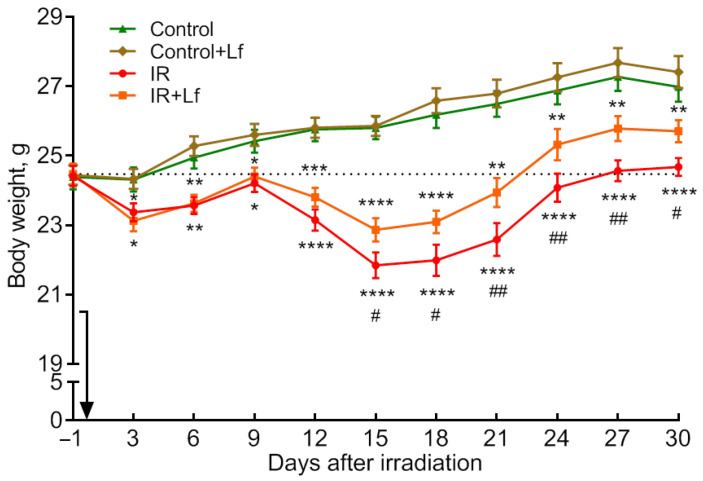
Effect of Lf on the body weight of mice after gamma-irradiation. The arrow shows the day of irradiation. *n* = 44/group (for IR and IR+Lf), *n* = 26/group (for Control and Control+Lf). The data are presented as mean ± SEM. * *p* < 0.05, ** *p* < 0.01, *** *p* < 0.001, **** *p* < 0.0001 in comparison with the corresponding control (sham-irradiated) groups on the same day; ^#^ *p* < 0.05, ^##^ *p* < 0.01—IR+Lf vs. IR on the same day.

The body weight of the animals in both control groups gradually increased. No differences were found between the Control and Control+Lf groups throughout the experiment (Figure 3).

The body weight of mice in the experimental groups (IR, IR+Lf) decreased on day 3 after irradiation and almost returned to the initial (before irradiation) level by day 9 (Figure 3). On days 12 and 15, the body weight decreased again, and this decrease was less pronounced in mice treated with Lf (day 15 IR+Lf vs. IR: *p* < 0.05). The irradiated animals gained weight starting from day 18. It should be noted that in the IR group, the highest number of deaths was recorded during the period from day 13 to day 18. The body weight of the animals in the experimental groups (IR, IR+Lf) differed from the control values from day 3 to the end of the experiment. However, the mice treated with Lf recovered faster by this parameter starting from day 15 (IR+Lf vs. IR: *p* < 0.05 for days 15, 18, 30; *p* < 0.01 for days 21, 24, 27) (Figure 3).

### 3.3. Lf Prevented Changes in the Behavior of Irradiated Mice

#### 3.3.1. Open Field Test

**Figure 4:**
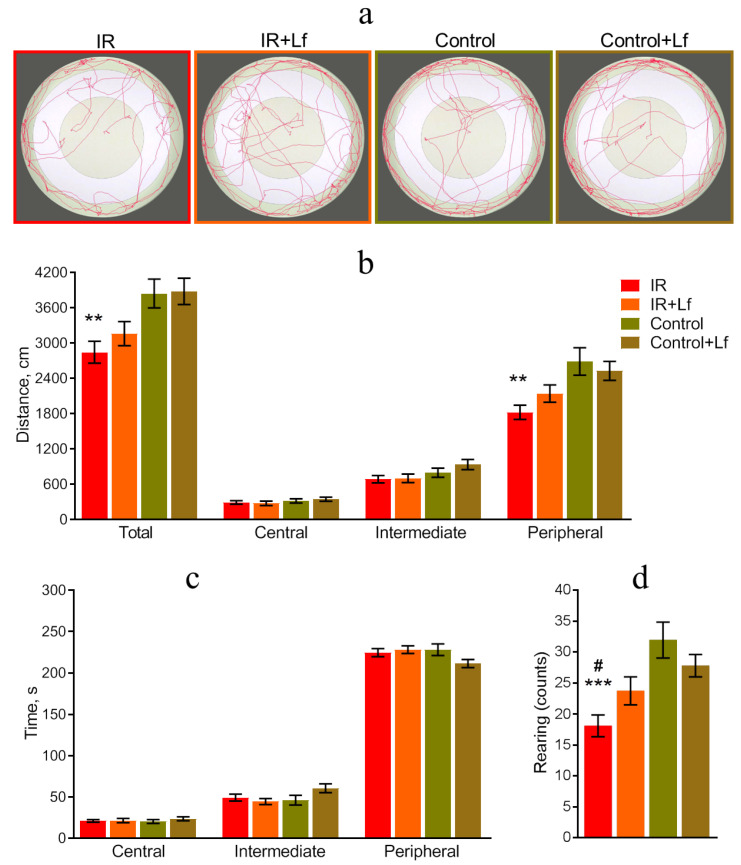
Effects of Lf on mouse behavior in the open field test after gamma-irradiation. *n* = 23 and 28 (for IR and IR+Lf), *n* = 14/group (for Control and Control+Lf). Representative 5 min movement tracks (**a**). Distance traveled in zones (**b**). Time spent in zones (**c**). Number of rearings (**d**). Values are presented as mean ± SEM. ** *p* < 0.01, *** *p* < 0.001 in comparison with the control group (Control); ^#^ *p* < 0.05 in comparison with the IR+Lf group.

The total distance traveled in the arena reflects the horizontal motor activity of mice [39]. The results of OF testing revealed reduced motor activity of animals of the IR group on day 26 after irradiation. Representative 5 min tracks of mouse movement in the OF are shown in Figure 4a. In the IR group, the total distance traveled and the distance traveled in the peripheral zone were shorter than in the control (*p* = 0.007 and *p* = 0.001, respectively) (Figure 4b). Irradiated animals treated with Lf (IR+Lf) did not differ significantly from the control (Control+Lf) by these parameters (Figure 4b).

The time spent by irradiated mice in each zone (central, intermediate, and peripheral) of the arena (Figure 4c) and the number of visits to the central zone (Control: 7.1 ± 0.7; Control+Lf: 7.3 ± 0.6; IR: 6.2 ± 0.6; IR+Lf: 6.0 ± 0.6) were similar to those in the control groups.

The number of rearings is an indicator reflecting exploratory activity of mice [40]. In irradiated animals (group IR), the number of rearings was reduced compared to both the control (Control; *p* = 0.0002) and irradiated animals treated with Lf (IR+Lf; *p* = 0.043) (Figure 4d). The number of rearings in irradiated and control mice treated with Lf did not differ (IR+Lf vs. Control+Lf: *p* = 0.522).

#### 3.3.2. Elevated Plus-Maze Testing

**Figure 5:**
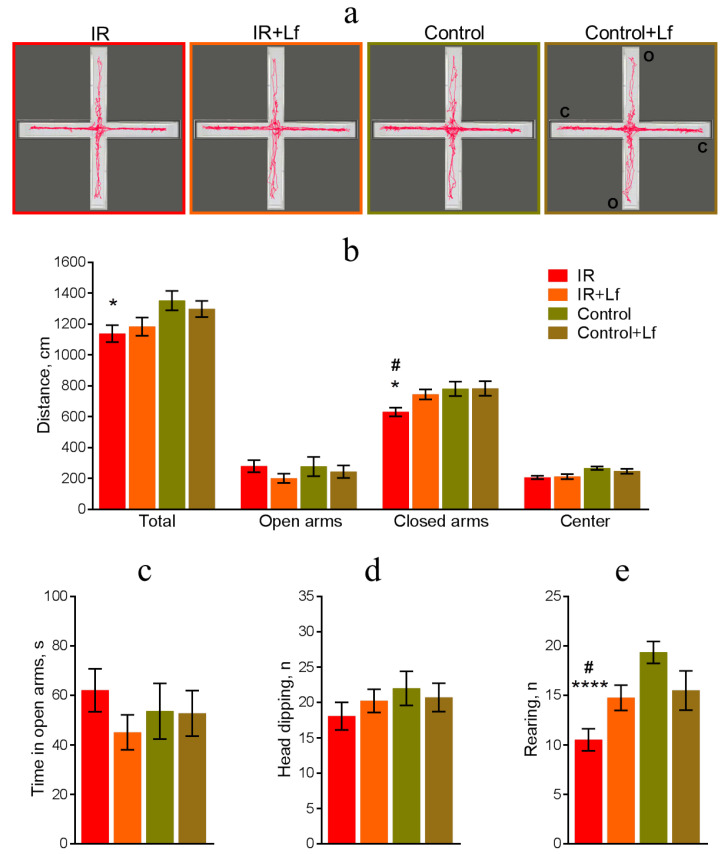
Effects of Lf on mouse behavior in the EPM test after gamma-irradiation. *n* = 23 and 28 (for IR and IR+Lf), *n* = 14/group (for Control and Control+Lf). Representative 5 min movement tracks (**a**); o—open arm; c—closed arm. Distance traveled (**b**). Time spent in open arms (**c**). Number of head dippings from open arms (**d**). Number of rearings (**e**). Values are presented as mean ± SEM. * *p* < 0.05, **** *p* < 0.0001 in comparison with the control group (Control); ^#^ *p* < 0.05 in comparison with the IR+Lf group.

The results of measuring the distance traveled in EPM on day 27 after irradiation also showed that the total motor activity of IR group mice was reduced. Representative 5 min tracks of mouse movement in the EPM are shown in Figure 5a. The total traveled path in the IR group was shorter than in the Control group (*p* = 0.034), and the path in closed arms of the maze was shorter than in the Control and IR+Lf groups (*p* = 0.02 and *p* = 0.043, respectively) (Figure 5b).

The mice of the experimental and control groups demonstrated similar behavior in the open arms of the maze: the path traveled in open arms (Figure 5b), the time spent in open arms (Figure 5c), and the number of head dippings (Figure 5d).

Irradiated animals (IR group) demonstrated lower number of rearings than in both the control (Control; *p* < 0.0001) and irradiated animals treated with Lf (IR+Lf; *p* = 0.043) (Figure 5e). The number of rearings was similar in the IR+Lf and Control+Lf groups (*p* = 0.977) (Figure 5e).

Thus, animals injected with Lf did not differ from controls by the studied parameters in the OF and EPM tests.

### 3.4. Dynamics of Blood Parameters in Irradiated Mice

The control groups (Control and Control+Lf) did not differ by the studied blood parameters throughout the experiment.

**Figure 6:**
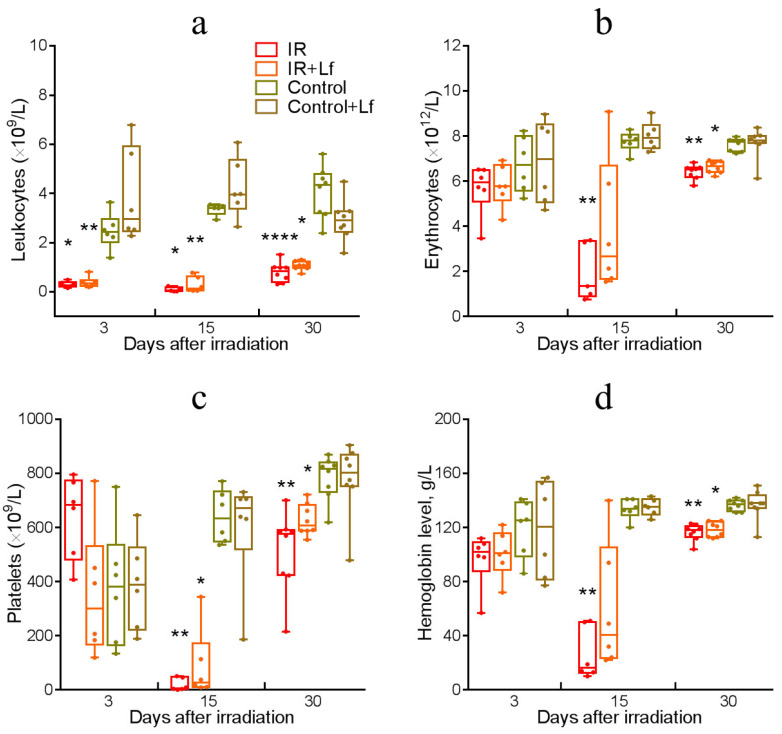
Dynamics of the number of leukocytes (**a**), erythrocytes (**b**), platelets (**c**) and hemoglobin level (**d**) in the blood of mice after gamma-irradiation. Data are presented as medians, quartiles, and minimum and maximum values. * *p* < 0.05, ** *p* < 0.01, **** *p* < 0.0001 in comparison with the corresponding control (sham-irradiated) groups on the same day.

Irradiation was followed by a sharp decrease in the blood leukocyte count in animals on day 3 (IR vs. Control: *p* = 0.017; IR+Lf vs. Control+Lf: *p* = 0.006) (Figure 6a). This parameter remained low in both experimental groups on day 15 (IR vs. Control: *p*= 0.034; IR+Lf vs. Control+Lf: *p*= 0.006) and day 30 after irradiation (IR vs. Control: *p* < 0.0001; IR+Lf vs. Control+Lf: *p* = 0.034).

In the experimental groups, a decrease in erythrocyte count (Figure 6b), platelet count (PLT, Figure 6c), and hemoglobin level (Figure 6d) was observed on day 15 (IR vs. Control: *p* = 0.006, 0.003, 0.005, respectively; IR+Lf vs. Control+Lf: *p* = 0.036, PLT). However, the irradiated mice receiving Lf did not differ from controls by the erythrocyte count and hemoglobin level (IR+Lf vs. Control+Lf: *p* = 0.136 and 0.118, respectively). These indicators greatly varied in animals of the IR+Lf group; in some mice, their values were close to control (Figure 6b,d). On day 30, erythrocyte and platelet count and hemoglobin levels in irradiated animals increased but still remained far below the control (IR vs. Control: *p* = 0.004, 0.002, 0.003, respectively; IR+Lf vs. Control+Lf: *p* = 0.017, 0.048, 0.011, respectively) (Figure 6b–d).

### 3.5. Dynamics of the Number of EdU^+^ Cells in the Bone Marrow of Irradiated Mice

**Figure 7:**
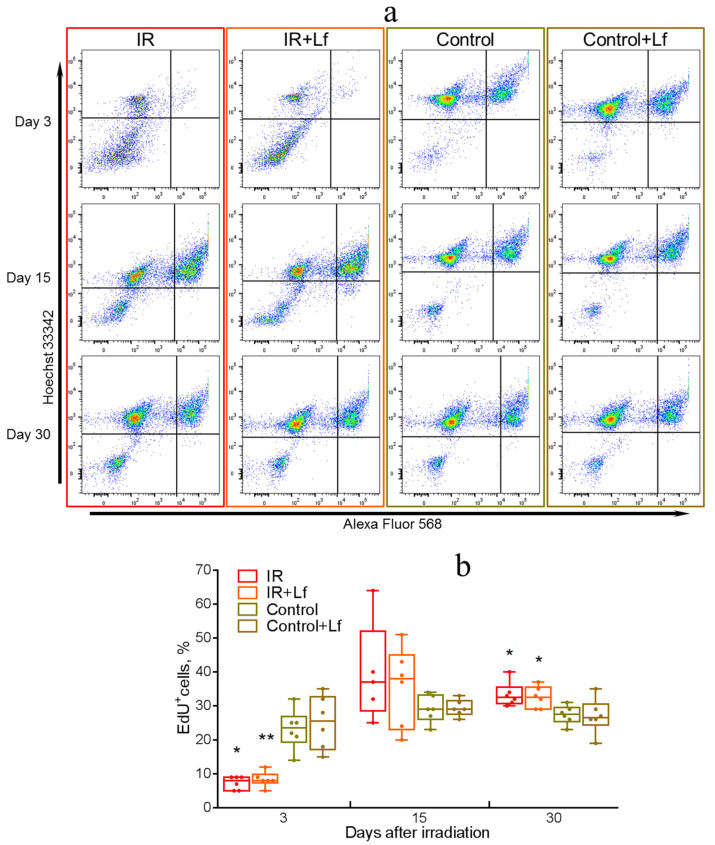
Flow cytometry analysis of the number of EdU^+^ cells in mouse bone marrow after whole-body gamma-irradiation. EdU (i.p.; 40 mg/kg) was administered 2 h before anesthesia. Incorporation of EdU into DNA was detected using a click reaction with Alexa Fluor 568 azide. Cell nuclei were poststained with Hoechst. Representative cytometric diagrams (**a**) for each group (columns) on days 3, 15, and 30 after irradiation (rows). Dynamics of the number of EdU^+^ cells throughout the experiment (%) (**b**). Data are presented as medians, quartiles, and minimum and maximum values. * *p* < 0.05, ** *p* < 0.01 in comparison with the corresponding control (sham-irradiated) groups on the same day.

The fraction of DNA-replicating cells was determined by incorporation of EdU (a synthetic thymidine analog) [41,42], which was detected by a click reaction with Alexa Fluor 568 azide. Representative cytometric diagrams are shown in Figure 7a.

Flow cytometry showed that the relative number of EdU^+^ cells in the bone marrow of control animals remained at a constant level throughout the experiment. A sharp decrease in this indicator was observed on day 3 after irradiation (IR vs. Control: *p* = 0.011; IR+Lf vs. Control+Lf: *p* = 0.008) (Figure 7a—upper row, b). In the experimental groups, the number of EdU^+^ cells increased significantly by day 15 (a wide intragroup variation was observed) (Figure 7a—middle row, b) and exceeded control levels on day 30 (IR vs. Control: *p* = 0.012; IR+Lf vs. Control+Lf: *p* = 0.029) (Figure 7a—bottom row, b). No differences were found between the irradiated groups (IR, IR+Lf) by the number of EdU^+^ cells in the bone marrow at these terms.

### 3.6. Radioprotective Effect of Lf on Mouse Brain Cells

#### 3.6.1. Proliferating (EdU^+^) Cells

**Figure 8:**
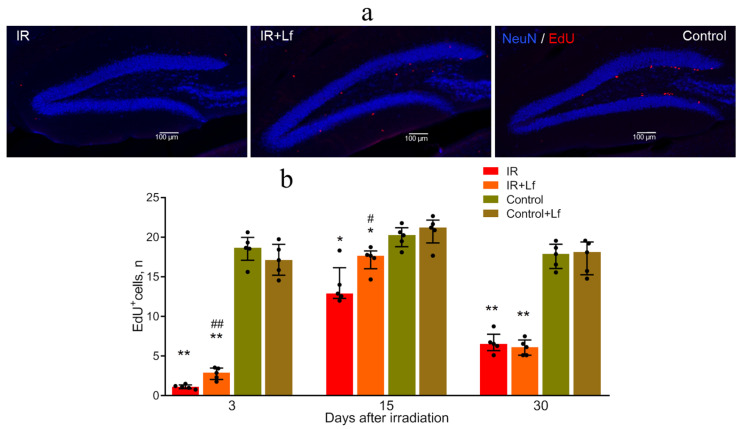
Immunofluorescent detection of NeuN protein (blue) and EdU incorporation (red) in cells of the dentate gyrus in mice subjected to whole-body gamma radiation. Representative micrographs of brain sections on day 3 (**a**). Quantitative analysis of EdU^+^ cells in the dentate gyrus of the hippocampus (**b**). * *p* < 0.05, ** *p* < 0.01 in comparison with the corresponding control (sham-irradiated) groups on the same day; ^#^ *p* < 0.05, ^##^ *p* < 0.01 in comparison with the IR group on the same day. Scale bars = 100 μm.

Analysis of stained brain sections showed that in control animals, the number of EdU^+^ cells in the subgranular zone of the hippocampal dentate gyrus remained at a constant level throughout the experiment (Figure 8b). On day 3 after irradiation, the number of EdU^+^ cells in this zone sharply decreased (IR vs. Control: *p* < 0.01; IR+Lf vs. Control+Lf: *p* < 0.01). After administration of Lf to irradiated animals, 2 times more EdU+ cells were preserved by this term (IR+Lf vs. IR: *p* < 0.01) (Figure 8a,b). After 15 days, a significant increase in this parameter was observed in both experimental groups. In irradiated animals treated with Lf, the number of proliferating cells was higher (IR+Lf vs. IR: *p* < 0.05) but still remained slightly below the control level (Figure 8b). On day 30, a decrease in the number of EdU^+^ cells was detected in both experimental groups (IR vs. Control: *p* < 0.01; IR+Lf vs. Control+Lf: *p* < 0.01).

#### 3.6.2. Immature Neurons (DCX^+^ Cells)

**Figure 9:**
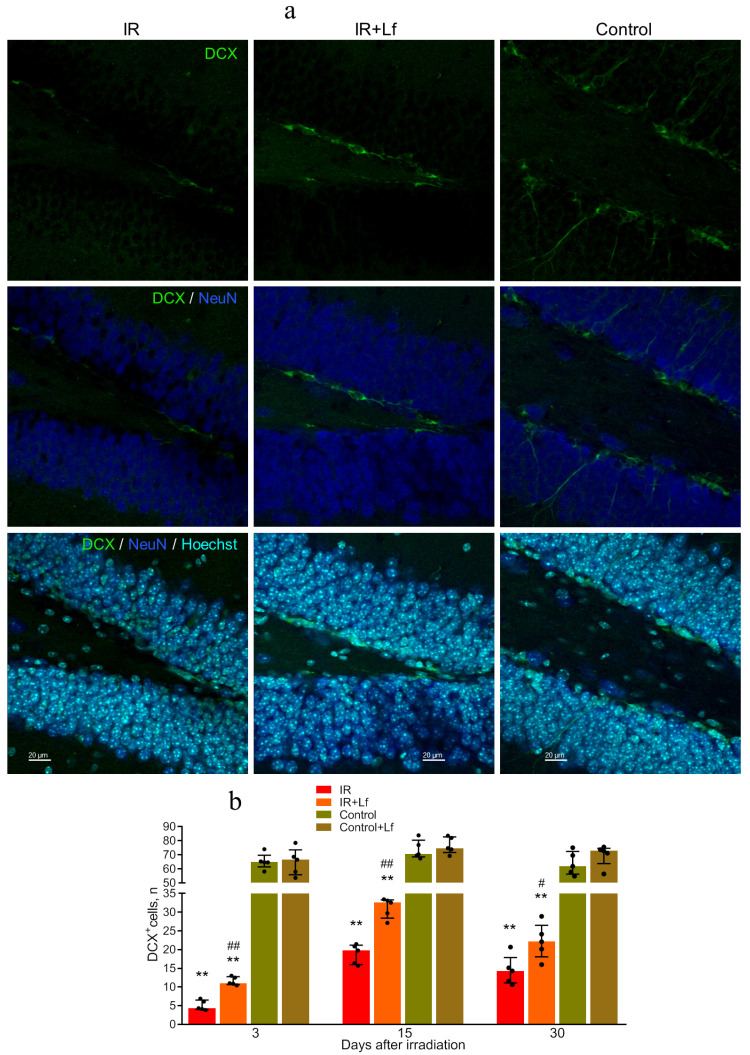
Immunofluorescent detection of DCX (green) and NeuN (blue) proteins in cells of the dentate gyrus in mice subjected to whole-body gamma radiation. Representative micrographs of brain sections on day 15. Nuclei were poststained with Hoechst (blue) (**a**). Quantitative analysis of DCX^+^ cells in the dentate gyrus of mouse hippocampus (**b**). ** *p* < 0.01 in comparison with the corresponding control (sham-irradiated) groups on the same day; ^#^ *p* < 0.05, ^##^ *p* < 0.01 in comparison with the IR group on the same day. Scale bars = 20 μm.

Immature neurons were labeled with an antibody against DCX, a protein associated with migration of neurons and neuroblasts [43]. In control mice, Lf had no appreciable effect on the number of DCX^+^ cells, and no differences were found between the Control and Control+Lf groups by this parameter throughout the experiment (Figure 9b). In irradiated animals, the number of DCX^+^ cells was significantly lower than in the control on days 3, 15, and 30 after exposure (*p* < 0.01) (Figure 9a,b). Lf treatment contributed to preservation of DCX^+^ cells; their number in the IR+Lf group was higher than in the IR group on days 3 (*p* < 0.01), 15 (*p* < 0.01), and 30 (*p* < 0.05) (Figure 9a,b).

**Figure 10:**
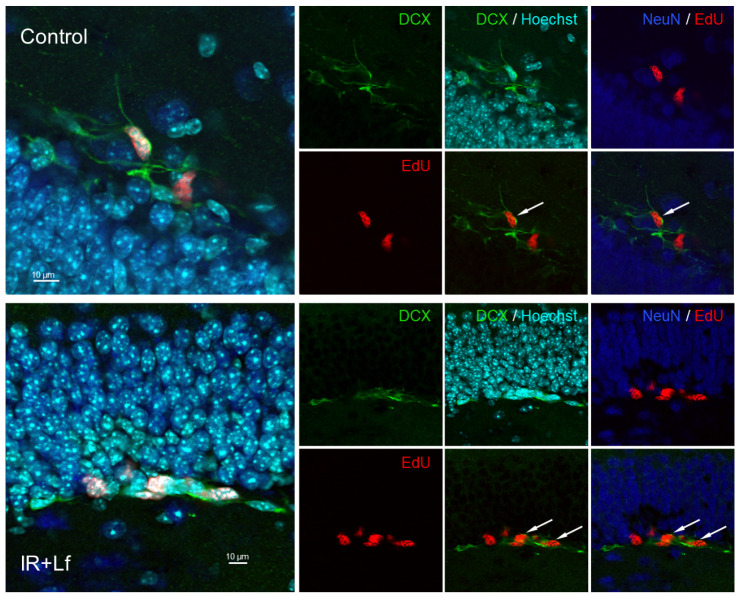
Immunofluorescent detection of EdU inclusion (red) and DCX (green) and NeuN (blue) proteins in cells of the dentate gyrus. Representative micrographs of brain sections from animals of the Control (**top**) and IR+Lf groups (**bottom**) on day 15 after gamma-irradiation. Nuclei are poststained with Hoechst (blue). Arrows show EdU^+^/DCX^+^ cells. Scale bars = 10 μm.

As expected, marker of mature neurons NeuN was not expressed in EdU^+^ cells. However, some of them were expressed DCX (Figure 10). Such EdU^+^/DCX^+^ cells were observed in the subgranular zone of the dentate gyrus in both control (on days 3, 15, and 30) and irradiated (on days 15 and 30) animals. In control (at all terms) and irradiated (on days 15 and 30) mice, EdU^+^ cells were often arranged in pairs or formed clusters (Figure 10).

## 4. Discussion

In this study, we evaluated the ability of Lf to mitigate radiation-induced disturbances in young adult mice subjected to whole-body gamma-irradiation at a dose of 7.5 Gy. While brain only irradiation studies are of help in determining direct effects of radiation on the brain, whole-body irradiation studies are more relevant in the context of space missions or catastrophic accidents, as they better reproduce radiation exposure [44]. In addition to the effects of radiation directly on the brain, whole-body irradiation also involves indirect effects of peripheral immune signals induced by the irradiation [45,46].

Modeling of acute radiation injury affects all aspects of the body’s functioning. The most obvious are significant body weight loss and a decrease in the mean life duration. It was shown that by day 14 after whole-body gamma-irradiation at a dose of 8 Gy, the body weight of mice decreased by 15% [29], and on day 30 after X-ray radiation at a dose of 6.8 Gy, the survival was 61.5% [17]. In our experiments, the irradiated animals (IR group) had lost about 11% of their body weight by day 15, and the survival rate by day 30 was 76.7%. Treatment with Lf (IR+Lf group) improved these indicators to 6% and 93.3%, respectively (Figure 2 and Figure 3). Massive animal death was recorded on days 13–18 in the IR group, but not in the irradiated group receiving Lf (IR+Lf) (Figure 2a). These data are consistent with our previous results [19,47].

Suppression of motor and exploratory activities was observed in rodents after gamma-irradiation at various doses [33,48,49,50]. The results of OF and EPM tests (on days 26 and 27 after the exposure, respectively) obtained by us for the IR group are consistent with the results of other researchers. In irradiated animals (IR group), the distance traveled in the OF and in closed EPM arms was shorter by 26 and 19%, respectively, and the number of rearings in both tests was lower by almost 2 times (44 and 46%, respectively) than in the control group. Lf treatment minimized changes in these behavioral parameters in irradiated mice (IR+Lf group) (Figure 4 and Figure 5). Open EPM arms are a mild stressor because of the risk of falling. More anxious mice tend to spend more time in closed arms and less often dip their heads from the open arms than less anxious animals [49,50]. Our experiments revealed no changes in anxiety in irradiated mice on day 27 after the exposure (Figure 5c,d).

Leukocytes are most sensitive to ionizing radiation among blood cells. Irradiation caused a sharp decrease in blood leukocyte count (on day 3), and this parameter did not recover until the end of the experiment (day 30) (Figure 6a). A decrease in the erythrocyte and platelet counts and hemoglobin levels was detected on day 15 (Figure 6b–d). Our data are in line with the results obtained by other authors [17,18,29]. In the IR+Lf group, these parameters greatly varied on day 15 after irradiation. In some animals, erythrocyte count and hemoglobin content were close to the control values. Intensive animal death from day 13 to day 18 was observed in the IR group but not in the irradiated group receiving Lf (IR+Lf) (Figure 2a). In the IR group, 10% of mice died by day 15 and 23% by day 30, while the corresponding death rates in the IR+Lf group were 0 and 7%. We believe that deaths of sick animals significantly contributed to improvement of blood parameters such as erythrocyte count and hemoglobin levels in the IR group. In our opinion, the early positive changes observed on day 15 in the IR+Lf group reflect specific effects of Lf rather than individual variability.

As the survival of mice irradiated at the sublethal dose depends on the recovery of the hematopoiesis system [29,51], we studied proliferative activity of bone marrow cells. For this purpose, 5-ethynyl-2′-deoxyuridine (EdU) was administered to animals. The incorporation of synthetic thymidine analogs into replicating DNA during the S-phase of the cell cycle is widely used for evaluating proliferative activity of cells in vivo [42,52,53]. Proliferating bone marrow cells are very sensitive to radiation and can be affected even at low doses. Irradiation led to a 3-fold decrease in the relative number of EdU^+^ cells in the bone marrow of animals on day 3 after the exposure. In the irradiated groups, this parameter increased and slightly exceeded the control levels on day 15; on day 30, it exceeded the control values by 16% (Figure 7). These data are consistent with the results obtained by other researchers. For instance, DNA content in the bone marrow significantly decreased in mice on day 3 after whole-body gamma-irradiation from a ^60^Co source at a dose of 3.5 Gy [54]. The total level of bone marrow cell apoptosis reached 21% 24 h after whole-body gamma-irradiation with ^60^Co at a dose of 8 Gy, on day 3 the total number of bone marrow cells decreased significantly, and by day 7 it doubled [29]. We found no differences between the irradiated groups in the number of proliferating cells in the bone marrow throughout the experiment.

The subgranular zone of the dentate gyrus of the hippocampus is an area of active cell proliferation in mammals [55,56]. Our previous studies and experiments performed by other researchers have demonstrated that radiation increased the level of apoptosis and inhibited proliferation and neurogenesis in this brain area [8,9,10,13,57]. Our previous study showed that acute gamma-irradiation of mouse head at a dose of 8 Gy depleted the population of immature neurons in the hippocampus [9]. In the present work, immature neurons were visualized with antibodies to DCX, a protein expressed in migrating neurons and neuroblasts. Irradiation led to a sharp decrease in the number of EdU^+^ and DCX^+^ cells (to 6 and 8% of the control group, respectively) in the dentate gyrus on day 3 after the exposure (Figure 8 and Figure 9). These data are in line with the results obtained by other authors [8,10,13]. On day 15, both indicators increased (to 70 and 26% of the control, respectively) and then decreased slightly again by day 30 (to 38 and 23% of the control, respectively). A transient increase in the number of proliferating cells in the dentate gyrus of adult rats on days 7 and 14 after cranial irradiation at doses of 2, 5, or 10 Gy has been reported [8]. This may reflect both changes in the cell proliferation rate and transition from asymmetric to symmetric division in the progenitor cell population. Lf administered to irradiated animals showed a protective effect on brain cells; it contributed to preservation and recovery of the population of both proliferating cells and immature neurons (DCX^+^ cells) (on day 3: 16 and 18% of the Control+Lf group, respectively; on day 15: 83 and 41% of the Control+Lf group, respectively) (Figure 8 and Figure 9).

Thus, a single injection of Lf to mice leads to at least a 2-fold increase in the number of proliferating cells and immature neurons in the dentate gyrus of the hippocampus in irradiated mice during the acute post-irradiation period (days 3 and 15) relatively to mice that did not receive Lf after irradiation. The current study is a continuation of our previous research focused on the short-term (within 6 h) effects of Lf after a single irradiation of mouse heads [9]. The long-term positive effects of Lf, to our knowledge, have been discovered for the first time in this study. We hypothesize that the protective effect of Lf on proliferating cells can be due to its antioxidant activity. Reactive oxygen species (ROS) and free radicals can initiate long-lasting alterative processes in various organs and tissues. In irradiated cells, ROS content can be considerably elevated due to shift of the oxidant–antioxidant balance and chronic inflammatory responses, thereby contributing to the long-term effects of ionizing radiation on genomic stability [58]. Under conditions of prolonged ROS generation, oxidative changes appear not only in irradiated cells but also in their progeny [59]. The destructive effects of free radicals can be prevented/mitigated by antioxidants. It is known that Lf is an antioxidant that inhibits the Fenton reaction through iron chelation [17].

Secondly, the increase in the number of proliferating cells in the dentate gyrus in irradiated mice under the influence of Lf may be a result of reprogramming of hippocampal cells susceptible to Lf into immature neurons. The biological effects of Lf are mediated by specific receptors on the surface of the target cells [60,61,62]. In our previous studies, immunohistochemical analysis revealed highly specific binding sites of exogenous Lf in the nuclei of neurons, astrocytes, and microglial cells in the mouse brain [63].

Recently, many studies have been conducted on how to compensate for the loss of neurons in damaged parts of the nervous system. It is known that in response to immunological problems or brain injuries, reactive astrocytes are activated and acquire neurogenic potential [64,65]. Ruggiero and colleagues [66] using *in vitro* cell culture models have demonstrated that Lf induced neurogenesis by promoting astrocytes reprogramming into immature neurons. Future studies are needed to examine whether Lf induces these processes in our model.

It is known that Lf can increase the levels of BDNF (brain neurotrophic factor) and the corresponding mRNA as well as the components of its signaling pathway in the hippocampus, reduce the expression of proinflammatory cytokines, suppress ROS generation [67,68], stimulate cell proliferation and differentiation [69], and enhance the expression of genes involved in the survival, differentiation, and growth of neurons [67,70]. The potential molecular mechanisms underlying the diverse effects of Lf are still under study. The mechanism of action of Lf, which contributes to preservation or accelerated recovery of the functional activity of mouse brain cells in our experimental model, seems to be related to interruption of one or several links in the pathogenesis of radiation-induced damage. The protein binds to the corresponding receptors, is internalized by endocytosis, and is translocated to the nucleus, where it modulates gene expression or triggers intracellular signaling pathways.

Some limitations of this study should be noted. OF and EPM tests used in the present study primarily assess general behavioral responses (general motor and exploratory activities and anxiety levels) rather than specific cognitive processes. In view of the revealed beneficial effects of Lf on the hippocampus at the cellular level, further experimental studies are needed to evaluate the potential of Lf in protecting against radiation-induced cognitive decline. To that end, we plan to use the Morris Water Maze test in the next phase of our research, because this test is strongly correlated with hippocampal function, which is crucial for spatial learning and memory.

## 5. Conclusions

The results of our study demonstrate a pronounced radio-mitigating effect of Lf in experimental animals with acute radiation-induced damage. Single administration of Lf improved animal survival during the experiment (30 days), compensated for irradiation-induced weight loss, prevented inhibition of motor and exploratory activity, and had a beneficial effect on the brain cells of irradiated mice. Our findings confirm the prospects for designing Lf-based radioprotective drugs for alleviation of the side effects of radiation therapy and for prevention and treatment of neurological complications of radiation injury.

## Data Availability

The original contributions presented in this study are included in the article. Further inquiries can be directed to the corresponding author. The raw data supporting the conclusions of this article will be made available by the authors on request.
